# Synthesis and Antibacterial Evaluation of Novel Heterocyclic Compounds Containing a Sulfonamido Moiety

**DOI:** 10.3390/molecules18010832

**Published:** 2013-01-11

**Authors:** Mohammad Emad Azab, Mohamed M. Youssef, Eman A. El-Bordany

**Affiliations:** 1 Organic Synthetic Lab, Chemistry Department, Faculty of Science, Ain Shams University, Cairo 11566, Egypt; 2 Department of Chemistry, Faculty of Science, Taif University, Taif 21974, Saudi Arabia

**Keywords:** cyanoacetylhydrazide, sulfonamide, pyridazines, pyrazoles, oxazole, pyrimidines, antibacterial activity

## Abstract

Aiming for the synthesis of new heterocyclic compounds containing a sulfonamido moiety suitable for use as antibacterial agents, the precursor ethyl {[4-*N*-(4,6-dimethylpyrimidin-2-yl)sulfamoyl]phenylazo}cyanoacetate was reacted with a variety of active methylene compounds producing pyran, pyridine and pyridazine derivatives. Also, the reactivity of the precursor hydrazone towards hydrazine derivatives to give pyrazole and oxazole derivatives was studied. On the other hand, treatment of the same precursor with urea, thiourea and/or guanidine hydrochloride furnished pyrimidine and thiazine derivatives, respectively. The newly synthesized compounds were tested for antibacterial activity, whereby eight compounds were found to have high activities.

## 1. Introduction

Simple nitrogen-containing heterocycles attached to sulfonamido moieties have received a large amount of attention in the literature, as a consequence of their exciting biological properties and their role as pharmacophores of considerable historical importance. Heterocyclic sulfonamides are used as carbonic anhydrase inhibitors [1,2,3], antibacterial agents [4], anticancer, anti-inflammatory and analgesic agents [5], β3-adrenergic receptor agonists [6], PC-1 inhibitors [7], antifungal agents [8] and antiviral agents [9]. For these vast biological activities and in continuation of our work [10,11,12,13,14,15,16,17,18] on the synthesis of novel heterocyclic systems exhibiting biological activity, we undertook the synthesis of a new series of compounds incorporating the abovementioned biologically active moieties in one molecule.

## 2. Results and Discussion 

### 2.1. Chemistry

**Scheme 1:**
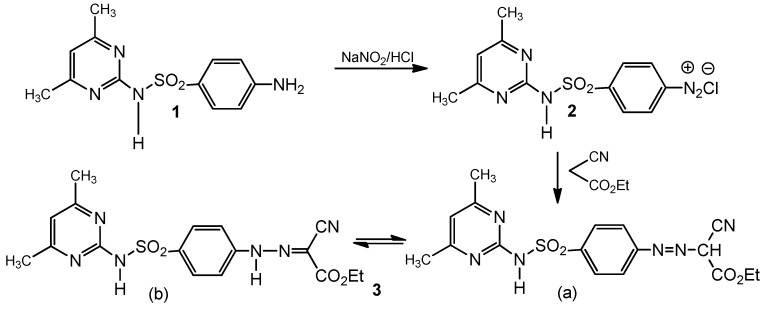
Diazotization and coupling of sulfamethazine; formation of **3**.

Synthesis of the precursor hydrazone **3** was achieved by diazotization of sulfamethazine, [*N*^1^-(4,6-dimethyl-2-pyrimidinyl)sulfanilamide, **1**] followed by coupling with ethyl cyanoacetate in the presence of sodium acetate at room temperature [19] (Scheme 1). The spectral data revealed that this compound exists in the hydrazone form (b), as the ^1^H-NMR spectrum showed two (exchangeable) signals at 6.91 and 9.12 corresponding to two NH groups and the MS indicated the molecular ion peak at 402, which is in accordance with the molecular formula.

**Scheme 2:**
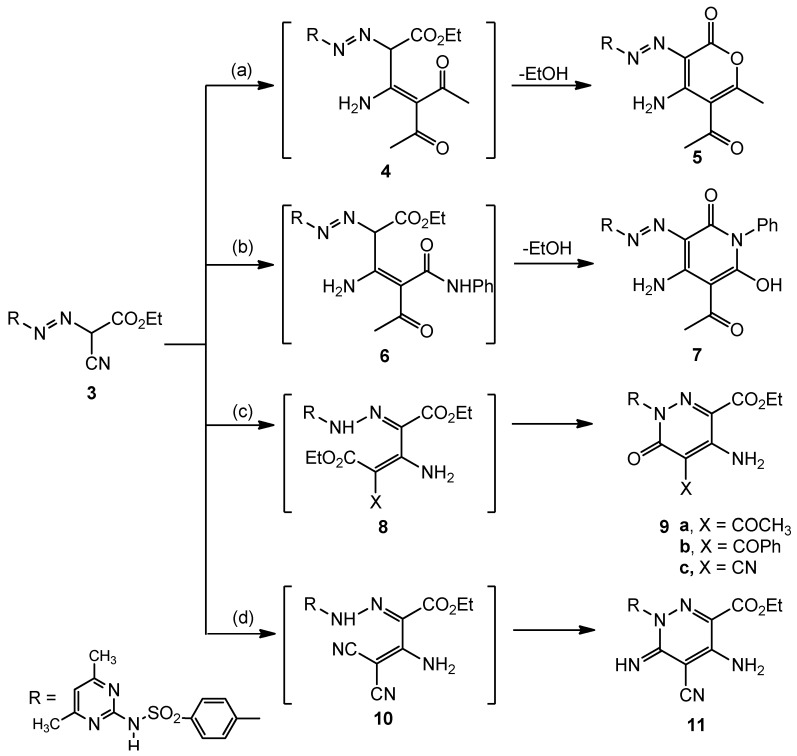
Cyclization of the arylazo cyanoacatate derivative **3** with active methylene compounds and β-dinitriles; formation of substituted pyrans, pyridines and pyridazines.

The reactivity of compound **3** towards active methylene reagents was investigated. Firstly, reaction of hydrazone **3** with dicarbonyl compounds was studied. Thus, when compound **3** reacted with acetylacetone in refluxing dioxane in the presence of catalytic amounts of triethylamine, the pyranone derivative **5** was obtained. Formation of **5** is believed to be formed via the intermediate **4** followed by the intramolecular cyclization with loss of an ethanol molecule. The structure of **5** was confirmed by the analytical and spectral data. Similarly, reaction of **3** with acetoacetanilide under the same reaction conditions afforded the pyridinone derivative **7** which is formed through the intermediate **6** followed by loss of an ethanol molecule (Scheme 2).

Secondly, the behavior of **3** with other active methylene compounds, such as ethyl acetoacetate, ethyl benzoylacetate, ethyl cyanoacetate and malononitrile, was investigated. This investigation resulted in the synthesis of polyfunctional substituted pyridazine derivatives **9a**–**c**, through the intermediate **8** and **11** through the intermediate **10**, respectively (Scheme 2). The structures of the synthesized compounds were elucidated based on their spectral data.

**Scheme 3:**
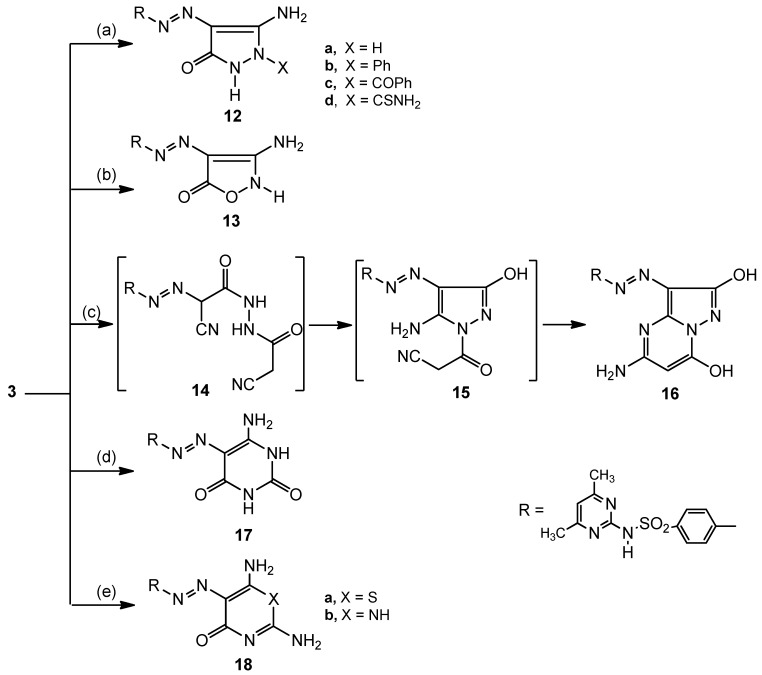
Cyclization of the arylazo cyanoacatate derivative **3** with amino compounds; formation of substituted pyrazoles, isoxazoles, pyrazolopyrimidines, pyrimidines and 1,3-thiazines.

The behaviour of **3** towards hydrazine derivatives was examined in order to prepare pyrazoles. Thus, treatment of **3** with hydrazine hydrate [20] (98%), phenylhydrazine, benzoylhydrazine or thiosemicarbazide [21] furnished the aminopyrazole derivatives **12a**–**d**, respectively. On the other hand, reaction of **3** with hydroxylamine hydrochloride in the presence of sodium acetate produced the isoxazole derivative **13** (Scheme 3). The structure of the prepared compounds were in accordance with their spectral data.

Pyrazolopyrimidine [22] derivative **16** was obtained when the hydrazone **3** was treated with cyanoacetohydrazide in refluxing dioxane in the presence of triethylamine. As a speculative mechanism for the formation of compound **16**, the intermediate **14** is firstly formed followed by an internal nucleophilic attack by the NH group on the cyano group. Then a migration of the two NH protons to the negatively charged nitrogen atom takes place to form the second intermediate **15**. Finally, **15** cyclized via nucleophilic attack by the NH_2_ group on the cyano group to produce the pyrazolopyrimidine derivative **16** (Scheme 3).

Treatment of compound **3** with urea, thiourea and guanidine in the presence of ethanolic sodium ethoxide [23] produces the pyrimidine and thiazine derivatives **17**, **18a**,**b** (Scheme 3). The formation of these compounds is assumed to be occur via the addition of the NH_2_ or SH groups to the cyano group followed by cyclization with elimination of an ethanol molecule.

### 2.2. Antibacterial Activity Evaluation

#### 2.2.1. Agar Diffusion Method

The obtained new compounds were screened* in vitro* for their antibacterial activities against Gram positive bacteria [*Staphylococcus aureus* (ATCC 25923) and *Bacillus cereus* (ATCC 10987)], Gram negative bacteria [*Serratia marcesens* (ATCC 274) and *Proteus mirabilis* (SM514)], using the agar diffusion technique. The results of the antibacterial activity tests are shown in Table 1.

Most of the synthesized compounds were found to possess some antibacterial activity towards all the microorganisms used. Compounds **3**, **12a**, **12b**, **12c**, **12d**, **16**, **17**, **18b** possess the highest antibacterial activities.

#### 2.2.2. Filter Paper Disc-Diffusion Method

The newly synthesized heterocyclic compounds listed in Table 2 were tested for their antibacterial activity against Gram positive bacteria [*Staphylococcus aureus* (ATCC 25923) and *Bacillus cereus* (ATCC 10987)], Gram negative bacteria [*Serratia marcesens* (ATCC 274) and *Proteus mirabilis* (SM514)]. The preliminary screening of the investigated compounds was performed using the filter paper disc-diffusion method. The most active compounds were **12a**, **12b**, **12d**, **16**, **17**, **18a** and **18b**, which were strongly inhibitory to all or some of the tested bacteria. Compounds **3**, **7** and **12c** showed moderate activities against the tested bacteria. The rest of compounds showed low or no sensitivity at all to the bacteria under investigation, and the results are summarized in Table 2.

## 3. Experimental

### 3.1. General

All melting points reported are uncorrected and were determined on a Stuart electric melting point apparatus. The microanalysis were within ±0.4% of theoretical values and were determined at the Microanalytical Unit of the Faculty of Science, Cairo University. The IR spectra were measured on a Perkin-Elmer 1600 FT-IR using the KBr wafer technique. The mass spectra were recorded on a Shimadzu GCMS-QP-1000EX mass spectrometer at 70 e.v. The ^1^H-NMR spectra were recorded in CDCl_3_ or DMSO-*d*_6_ solutions on a Bruker 200 MHz instrument using TMS as internal standard with chemical shifts expressed in ppm.^ 13^C-NMR spectra were recorded on a Varian Mercury 300 MHz spectrometer using TMS as an internal standard and DMSO-*d*_6_ as solvent. TLC was performed on ready-to-use Merck 60 silica gel plates to monitor the reactions and test the purity of the new synthesized compounds.

#### 3.1.1. Ethyl {[4-*N*-(4,6-dimethylpyrimidin-2-yl)sulfamoyl]phenylazo}cyanoacetate (**3**)

This compound was prepared according to a previously reported method [19]. Yellow solid (from ethanol), yield 81%, m.p. 228–230 °C. IR (ν/cm^−1^): 3232, 3192 (NH), 3053 (CH_ar_), 2989 (CH_al_), 2221 (C≡N), 1723 (C=O), 1610 (C=N), 1563 (N=N), 1327, 1150 cm^−1^ (SO_2_). ^1^H-NMR (DMSO-*d_6_*): 1.22 (t, 3H, OCH_2_CH_3_, *J* = 4.4 Hz), 2.21 (s, 6H, 2 CH_3_), 4.27 (q, 2H, OCH_2_CH_3_, *J* = 4.4 Hz), 6.91 (s, 1H, NH, D_2_O exchangeable), 7.57–8.15 (m, 5H, Ar-H + pyrimidine-H), 9.12 (s, 1H, NH, D_2_O exchangeable). MS (%): molecular ion peak at 402 (3.5) and M+1 at 403 (4.5). C_17_H_18_N_6_O_4_S (402); Calcd: C, 50.74; H, 4.51; N, 20.88; S, 7.97; Found: C, 51.00; H, 4.70; N, 21.10; S, 7.80.

#### 3.1.2. Reaction of **3** with Acetylacetone or Acetoacetanilide; Formation of **5** and **7**

A mixture of **3** (5 mmol, 2.06 g) and acetylacetone or acetoacetanilide (5 mmol) was dissolved in dioxane (40 mL) containing triethylamine (5 drops). The reaction mixture was heated under reflux for 6–8 h and concentrated. The solid product that precipitated after cooling was filtered of and crystallized from the proper solvent to compound **5** and **7**, respectively.

*5-Acetyl-4-amino-3-{[4-N-(4,6-dimethylpyrimidin-2-yl)sulfamoyl]phenylazo}-6-methyl-2H-pyran-2-one* (**5**): Pale yellow solid (from dioxane), yield 68% (1.55 g), m.p. 263–265 °C. IR (ν/cm^−1^): 3433, 3364, 3224, 3171 (NH_2_, NH), 2996, 2943 (CH), 1689, 1661 (C=O) cm^−1^. ^1^H-NMR (DMSO-*d_6_*): 2.21 (s, 6H, 2 CH_3_), 2.47 (s, 3H, CH_3_), 2.68 (s, 3H, COCH_3_), 5.66(s, 2H, NH_2_, D_2_O exchangeable), 6.95 (s, 1H, NH, D_2_O exchangeable), 7.51–8.22 (m, 5H, Ar-H + pyrimidine-H). ^13^C-NMR (DMSO-*d_6_*): 20.3 (CH_3_), 25.1 (2CH_3_), 35.6 (CH_3_CO), 113.4, 118.5, 119.3, 121.7, 122.3, 124.8, 125.5, 128.4, 131.6, 133.9, 138.4 (Ar-C), 166.4, 181.5 (CO). MS (%): molecular ion peak at 456 (3.5) and M+1 at 457 (4.5). C_20_H_20_N_6_O_5_S (456); Calcd: C, 52.62; H, 4.42; N, 18.41; S, 7.02; Found: C, 52.40; H, 4.70; N, 18.20; S, 7.30.

*5-Acetyl-4-amino-3-{[4-N-(4,6-dimethylpyrimidin-2-yl)sulfamoyl]-phenylazo}-6-hydroxy-1-phenylpyridin-2(1H)-one* (**7**): Yellow solid (from dioxane), yield 60% (1.60 g), m.p. 271–274 °C. IR (ν/cm^−1^): 3502, 3435, 3364, 3228, 3176 (OH, NH_2_, NH), 2996, 2943 (CH), 1691, 1660 (C=O) cm^−1^. ^1^H-NMR (DMSO-*d_6_*): 2.23 (s, 6H, 2 CH_3_), 2.64 (s, 3H, COCH_3_), 5.78(s, 2H, NH_2_, D_2_O exchangeable), 6.94 (s, 1H, NH, D_2_O exchangeable), 7.23–8.12 (m, 10H, Ar-H + pyrimidine-H), 10.34 (s, 1H, OH, D_2_O exchangeable). ^13^C-NMR (DMSO-*d_6_*): 25.1 (2CH_3_), 35.5 (CH_3_CO), 114.1, 116.8, 119.2, 120.0, 120.7, 122.3, 124.5, 125.6, 128.3, 129.7, 132.1, 134.3, 140.6, 141.4, 143.1 (Ar-C), 161.7, 184.1 (CO). MS (%): molecular ion peak at 533 (2.7). C_25_H_23_N_7_O_5_S (533); Calcd: C, 56.28; H, 4.34; N, 18.38; S, 6.01; Found: C, 56.00; H, 4.60; N, 15.20; S, 6.10.

#### 3.1.3. Reaction of **3** with β-ketoesters, β-cyanoesters and β-dicarbonitriles; Formation of **9a**–**c** and **11**

Equimolar amounts of **3** (5 mmol, 2.06 g) and an active methylene compound, namely ethyl acetoacetate, ethyl benzoylacetate, ethyl cyanoacetate or malononitrile (5 mmol) in dioxane (40 mL) containing triethylamine (5 drops), were heated under reflux for 8–10 h. The solid product so obtained on cooling was collected by filtration and crystallized from the appropriate solvent to give compounds **9a**–**c** and **11**, respectively.

*Ethyl 5-acetyl-4-amino-1-[4-N-(4,6-dimethylpyrimidin-2-yl)sulfamoyl]phenyl-1,6-dihydro-6-oxopyrid-azine-3-carboxylate* (**9a**): Yellow solid (from dioxane), yield 51% (1.24 g), m.p. 260–263 °C. IR (ν/cm^−1^): 3443, 3356, 3229, 3169 (NH_2_, NH), 1728, 1686, 1666 (C=O) cm^−1^. ^1^H-NMR (DMSO-*d_6_*): 1.29 (t, 3H, OCH_2_CH_3_, *J* = 4.3 Hz), 2.24 (s, 6H, 2 CH_3_), 2.68 (s, 3H, COCH_3_), 4.25 (q, 2H, OCH_2_CH_3_, *J* = 4.3 Hz), 5.76(s, 2H, NH_2_, D_2_O exchangeable), 6.89 (s, 1H, NH, D_2_O exchangeable), 7.49–8.14 (m, 5H, Ar-H + pyrimidine-H). ^13^C-NMR (DMSO-*d_6_*): 15.2 (CH_3_), 25.1 (2CH_3_), 35.5 (CH_3_CO), 63.2 (CH_2_), 110.1, 117.3, 119.4, 120.3, 126.5, 129.7, 132.4, 134.1, 139.1, 142.7 (Ar-C), 161.5, 168.4, 183.9 (CO). MS (%): molecular ion peak at 486 (22.4). C_21_H_22_N_6_O_6_S (486); Calcd: C, 51.84; H, 4.56; N, 17.27; S, 6.59; Found: C, 52.00; H, 4.70; N, 16.90; S, 6.80.

*Ethyl 4-amino-5-benzoyl-1-[4-N-(4,6-dimethylpyrimidin-2-yl)sulfamoyl]phenyl-1,6-dihydro-6-oxo-pyridazine-3-carboxylate* (**9b**): Orange solid (from dioxane-water), yield 52% (1.43 g), m.p. 272–275 °C. IR (ν/cm^−1^): 3435, 3345, 3230, 3173 (NH_2_, NH), 2989, 2941 (CH_3_), 1730, 1688, 1662 (C=O) cm^−1^. ^1^H-NMR (DMSO-*d_6_*): 1.30 (t, 3H, OCH_2_CH_3_, *J* = 4.4 Hz), 2.22 (s, 6H, 2 CH_3_), 4.23 (q, 2H, OCH_2_CH_3_, *J* = 4.4 Hz), 5.81 (s, 2H, NH_2_, D_2_O exchangeable), 6.92 (s, 1H, NH, D_2_O exchangeable), 7.33–8.17 (m, 10H, Ar-H + pyrimidine-H). MS (%): molecular ion peak at 548 (13.1). C_26_H_24_N_6_O_6_S (548); Calcd: C, 56.93; H, 4.41; N, 15.32; S, 5.85; Found: C, 57.10; H, 4.70; N, 15.10; S, 5.60.

*Ethyl 4-amino-5-cyano-1-[4-N-(4,6-dimethylpyrimidin-2-yl)sulfamoyl]phenyl-1,6-dihydro-6-oxopyrid-azine-3-carboxylate* (**9c**): Yellow solid (from dioxane), yield 59% (1.38 g), m.p. 281–284 °C. IR (ν/cm^−1^): 3445, 3349, 3240, 3179 (NH_2_, NH), 2218 (C≡N), 1728, 1690 (C=O) cm^−1^. ^1^H-NMR (DMSO-*d_6_*): 1.28 (t, 3H, OCH_2_CH_3_, *J* = 4.4 Hz), 2.26 (s, 6H, 2 CH_3_), 4.28 (q, 2H, OCH_2_CH_3_, *J* = 4.4 Hz), 5.78 (s, 2H, NH_2_, D_2_O exchangeable), 6.94 (s, 1H, NH, D_2_O exchangeable), 7.52–8.11 (m, 5H, Ar-H + pyrimidine-H). MS (%): molecular ion peak at 469 (18.1). C_20_H_19_N_7_O_5_S (469): Calcd; C, 51.17; H, 4.08; N, 20.88; S, 6.83; Found: C, 51.30; H, 3.80; N, 21.10; S, 7.00.

*Ethyl 4-amino-5-cyano-1-[4-N-(4,6-dimethylpyrimidin-2-yl)sulfamoyl]phenyl-1,6-dihydro-6-iminopy-ridazine-3-carboxylate* (**11**): Light brown solid (from acetic acid), yield 71% (1.38 g), m.p. 293–296 °C. IR (ν/cm^−1^): 3430, 3339, 3235, 3176 (NH_2_, NH), 2220 (C≡N), 1726 (C=O) cm^−1^. ^1^H-NMR (DMSO-*d_6_*): 1.30 (t, 3H, OCH_2_CH_3_, *J* = 4.4 Hz), 2.27 (s, 6H, 2 CH_3_), 4.22 (q, 2H, OCH_2_CH_3_, *J* = 4.4 Hz), 5.67 (s, 2H, NH_2_, D_2_O exchangeable), 6.88 (s, 1H, NH, D_2_O exchangeable), 7.42–8.16 (m, 5H, Ar-H + pyrimidine-H), 10.88 (br s, 1 H, =NH, D_2_O exchangeable). MS (%): molecular ion peak at 468 (24.8). ^13^C-NMR (DMSO-*d_6_*): 15.3 (CH_3_), 25.1 (2CH_3_), 63.4 (CH_2_), 115.3 (CN), 118.1, 119.3, 120.8, 121.4, 125.3, 127.1, 129.7, 132.4, 135.1, 139.7, 153.3 (Ar-C), 164.3 (CO). MS (%): molecular ion peak at 468 (19.5). C_20_H_20_N_8_O_4_S (468); Calcd: C, 51.27; H, 4.30; N, 23.92; S, 6.84; Found: C, 51.50; H, 4.10; N, 24.10; S, 7.10.

#### 3.1.4. Reaction of 3 with Diamino Compounds; Formation of **12a**–**d**

To a solution of **3** (5 mmol, 2.06 g) in dioxane (40 mL), hydrazine hydrate (98%), phenylhydrazine, benzoylhydrazine or thiosemicarbazide (5 mmol, 0.25 mL) was added and the reaction mixture was refluxed for 2–6 h. The solid product which formed on heating was collected and crystallized from the proper solvent to afford compounds **12a**–**d**, respectively.

*5-Amino-4-{[4-N-(4,6-dimethylpyrimidin-2-yl)sulfamoyl]phenylazo}-pyrazol-5(1H,2H)-one* (**12a**): Brown solid, (from acetic acid), yield 79% (1.53 g), m.p. 261–263 °C. IR (ν/cm^−1^): 3444, 3351, 3239, 3171 (NH_2_, NH), 1677 (C=O), 1626 (C=N) cm^−1^. ^1^H-NMR (DMSO-*d_6_*): 2.25 (s, 6H, 2 CH_3_), 5.85 (s, 2H, NH_2_, D_2_O exchangeable), 6.87 (s, 1H, NH, D_2_O exchangeable), 7.42–8.19 (m, 5H, Ar-H + pyrimidine-H), 8.99, 10.63 (br s, 2 H, 2NH, D_2_O exchangeable). ^13^C-NMR (DMSO-*d_6_*): 24.1 (2CH_3_), 101.1, 110.3, 117.2, 119.8, 121.4, 123.6, 128.1, 133.5, 140.3 (Ar-C), 164.9 (CO). MS (%): molecular ion peak at 388 (36.8). C_15_H_16_N_8_O_3_S (388); Calcd: C, 46.38; H, 4.15; N, 28.85; S, 8.26; Found: C, 46.60; H, 3.90; N, 29.10; S, 8.10.

*5-Amino-4-{[4-N-(4,6-dimethylpyrimidin-2-yl)sulfamoyl]phenylazo}-1-phenylpyrazol-5(2H)-one* (**12b**): Light brown solid, (from acetic acid), yield 63% (1.46 g), m.p. 254–256 °C. IR (ν/cm^−1^): 3435, 3346, 3231, 3167 (NH_2_, NH), 1679 (C=O), 1619 (C=N) cm^−1^. ^1^H-NMR (DMSO-*d_6_*): 2.24 (s, 6H, 2 CH_3_), 5.90 (s, 2H, NH_2_, D_2_O exchangeable), 6.86 (s, 1H, NH, D_2_O exchangeable), 7.32–8.13 (m, 10H, Ar-H + pyrimidine-H), 10.61 (s, 1H, NH, D_2_O exchangeable). C_21_H_20_N_8_O_3_S (464); Calcd: C, 54.30; H, 4.34; N, 24.12; S, 6.90; Found: C, 54.50; H, 4.10; N, 24.30; S, 7.10.

*5-Amino-1-benzoyl-4-{[4-N-(4,6-dimethylpyrimidin-2-yl)sulfamoyl]-phenylazo}pyrazol-5(2H)-one* (**12c**): Brown solid, (from acetic acid), yield 56% (1.38 g), m.p. 248–250 °C. IR (ν/cm^−1^): 3439, 3342, 3221, 3174 (NH_2_, NH), 1680, 1652 (C=O), 1617 (C=N) cm^−1^. ^1^H-NMR (DMSO-*d_6_*): 2.25 (s, 6H, 2 CH_3_), 5.93 (s, 2H, NH_2_, D_2_O exchangeable), 6.89 (s, 1H, NH, D_2_O exchangeable), 7.39–8.13 (m, 10H, Ar-H + pyrimidine-H), 10.66 (s, 1H, NH, D_2_O exchangeable). C_22_H_20_N_8_O_4_S (492); Calcd: C, 53.65; H, 4.09; N, 22.75; S, 6.51; Found: C, 53.80; H, 3.90; N, 22.50; S, 6.30.

*5-Amino-1-carbothioamido-4-{[4-N-(4,6-dimethyl-pyrimidin-2-yl)sulfamoyl]phenylazo}-pyrazol-5(2H)-one* (**12d**): Brown solid, (from acetic acid), yield 66% (1.47 g), m.p. 277–280 °C. IR (ν/cm^−1^): 3438, 3337, 3212, 3162 (NH_2_, NH), 1678 (C=O), 1615 (C=N) cm^−1^. ^1^H-NMR (DMSO-*d_6_*): 2.21 (s, 6H, 2 CH_3_), 5.82 (s, 2H, NH_2_, D_2_O exchangeable), 6.26 (s, 2H, NH_2_, D_2_O exchangeable), 6.96 (s, 1H, NH, D_2_O exchangeable), 7.48–8.19 (m, 5 H, Ar-H + pyrimidine-H), 10.65 (s, 1H, NH, D_2_O exchangeable). C_16_H_17_N_9_O_3_S_2_ (447); Calcd: C, 42.94; H, 3.83; N, 28.17; S, 14.33; Found: C, 43.20; H, 4.00; N, 27.90; S, 14.10.

#### 3.1.5. 3-Amino-4-{[4-*N*-(4,6-dimethylpyrimidin-2-yl)sulfamoyl]phenylazo}-isoxazol-5(2*H*)one (**13**)

To dioxane (40 mL) containing sodium acetate (0.5 g), the hydrazone **3** (5 mmol, 2.06 g) and hydroxylamine hydrochloride (5 mmol, 0.35 g) were added. The mixture was refluxed for 8 h., left to cool then poured onto ice/water. The solid product so formed was filtered off, dried and crystallized from dioxane-water as pale brown solid, yield 61% (1.21 g), m.p. 267–269 °C. IR (ν/cm^−1^): 3434, 3339, 3218, 3164 (NH_2_, NH), 1679 (C=O), 1616 (C=N) cm^−1^. ^1^H-NMR (DMSO-*d_6_*): 2.22 (s, 6H, 2 CH_3_), 5.88 (s, 2H, NH_2_, D_2_O exchangeable), 6.94 (s, 1H, NH, D_2_O exchangeable), 7.52–8.21 (m, 5 H, Ar-H + pyrimidine-H), 10.77 (s, 1H, NH, D_2_O exchangeable). ^13^C-NMR (DMSO-*d_6_*): 24.1 (2CH_3_), 98.3, 110.1, 117.2, 119.3, 121.4, 123.6, 128.1, 138.5, 145.6 (Ar-C), 173.0 (CO). MS (%): molecular ion peak at 389 (21.3). C_15_H_15_N_7_O_4_S (389); Calcd: C, 46.27; H, 3.88; N, 25.18; S, 8.23; Found: C, 46.10; H, 4.10; N, 24.90; S, 8.10.

#### 3.1.6. 5-Amino-3-{[4-*N*-(4,6-dimethylpyrimidin-2-yl)sulfamoyl]phenylazo}-2,7-dihydroxypyrazolo-[1,5-a]pyrimidine (**16**)

Compound **3** (5 mmol, 2.06 g) and cyanoacetohydrazide (5 mmol, 0.45 g) were dissolved in dioxane (40 mL) containing a few drops of triethylamine. The mixture was refluxed for 19 h., left to cool then poured onto dil.HCl/ice. The solid product precipitated was filtered off, dried and crystallized from dioxane as orange solid, yield 55% (1.25 g), m.p. 291–293 °C. IR (ν/cm^−1^): 3503, 3423, 3331, 3221, 3169 (OH, NH_2_, NH), 1619 (C=N) cm^−1^. ^1^H-NMR (DMSO-*d_6_*): 2.21 (s, 6H, 2 CH_3_), 5.86 (s, 2H, NH_2_, D_2_O exchangeable), 6.92 (s, 1H, NH, D_2_O exchangeable), 7.42–8.27 (m, 6H, Ar-H + 2pyrimidine-H), 10.88 (s, 1H, OH, D_2_O exchangeable), 11.43 (s, 1H, OH, D_2_O exchangeable) ^13^C-NMR (DMSO-*d_6_*): 24.1 (2CH_3_), 103.4, 111.7, 117.3, 120.1, 122.3, 123.7, 128.0, 130.4, 137.2, 139.3, 141.4, 145.1 (Ar-C). MS (%): molecular ion peak at 455 (13.9). C_18_H_17_N_9_O_4_S (455); Calcd: C, 47.47; H, 3.76; N, 27.68; S, 7.04; Found: C, 47.20; H, 4.00; N, 27.90; S, 6.90.

#### 3.1.7. Reaction of 3 with Urea, Thiourea & Guanidine; Formation of **17**, **18a**,**b**

To a mixture of **3** (5 mmol, 1.42 g) and the proper amino compound (6 mmol) (urea thiourea or guanidine) in absolute ethanol (40 mL), sodium ethoxide (0.23 g of Na in 10 mL ethanol) was added. The reaction mixture was refluxed for 6–8 h, concentrated and cooled. The separated solid was filtered off, washed with water several times and crystallized from the proper solvent to afford compounds **17**,**18a**,**b**, respectively.

*6-Amino-5-{[4-N-(4,6-dimethylpyrimidin-2-yl)sulfamoyl]phenylazo}-pyrimidine-2,4-(1H,3H)-dione* (**17**): Yellow solid (from dioxane), yield 77% (1.47 g), m.p. 270–272 °C. IR (ν/cm^−1^): 3448, 3347, 3212, 3162 (NH_2_, NH), 1670 (C=O) cm^−1^. ^1^H-NMR (DMSO-*d_6_*): 2.27 (s, 6H, 2 CH_3_), 5.62 (s, 2H, NH_2_, D_2_O exchangeable), 6.87 (s, 1H, NH, D_2_O exchangeable), 7.56–8.18 (m, 5H, Ar-H + pyrimidine-H), 9.41–9.89 (br, 2H, 2NH, D_2_O exchangeable). ^13^C-NMR (DMSO-*d_6_*): 24.1 (2CH_3_), 95.3, 110.1, 118.7, 120.4, 123.1, 126.3, 131.6, 135.2, 142.9 (Ar-C), 159.3, 162.7 (CO). MS (%): molecular ion peak at 416 (11.2). C_16_H_16_N_8_O_4_S (416); Calcd: C, 46.15; H, 3.87; N, 26.91; S, 7.70; Found: C, 46.40; H, 3.70; N, 27.10; S, 7.90.

*2,6-Diamino-5-{[4-N-(4,6-dimethylpyrimidin-2-yl)sulfamoyl]phenyl-azo}-4H-1,3-thiazin-4-one* (**18a**): Orange solid (from dioxane), yield 69% (1.47 g), m.p. 296–299 °C. IR (ν/cm^−1^): 3441, 3355, 3225, 3176 (NH_2_, NH), 1667 (C=O) cm^−1^. ^1^H-NMR (DMSO-*d_6_*): 2.25 (s, 6H, 2CH_3_), 5.33 (s, 2H, NH_2_, D_2_O exchangeable), 5.65 (s, 2H, NH_2_, D_2_O exchangeable), 6.84 (s, 1H, NH, D_2_O exchangeable), 7.51–8.23 (m, 5H, Ar-H + pyrimidine-H). ^13^C-NMR (DMSO-*d_6_*): 24.1 (2CH_3_), 107.9, 110.1, 119.3, 120.8, 123.2, 125.0, 130.1, 136.0, 143.9, 151.2 (Ar-C), 167.4 (CO). MS (%): molecular ion peak at 432 (7.7). C_16_H_16_N_8_O_3_S_2_ (432); Calcd: C, 44.43; H, 3.73; N, 25.91; S, 14.83; Found: C, 44.20; H, 3.80; N, 26.10; S, 15.00.

*2,6-Diamino-5-{[4-N-(4,6-dimethylpyrimidin-2-yl)sulfamoyl]phenylazo}-pyrimidin-4(1H)-one* (**18b**): Orange solid (from dioxane), yield 61% (1.27 g), m.p. 261–263 °C. IR: 3433, 3364, 3189 (NH_2_, NH), 1669 (C=O) cm^−1^. ^1^H-NMR (DMSO-*d_6_*): 2.29 (s, 6H, 2 CH_3_), 5.38 (s, 2H, NH_2_, D_2_O exchangeable), 5.67 (s, 2H, NH_2_, D_2_O exchangeable), 6.89 (s, 1H, NH, D_2_O exchangeable), 7.43–8.24 (m, 5H, Ar-H + pyrimidine-H), 9.83 (br., 1H, NH, D_2_O exchangeable).^ 13^C-NMR (DMSO-*d_6_*): 24.1 (2CH_3_), 92.9, 110.1, 118.5, 120.1, 121.7, 125.4, 130.1, 135.5, 141.3, 153.0 (Ar-C), 162.1 (CO). MS (%): molecular ion peak at 415 (14.5). C_16_H_17_N_9_O_3_S (415); Calcd: C, 46.26; H, 4.12; N, 30.34; S, 7.72; Found: C, 46.10; H, 3.50; N, 30.10; S, 7.60.

### 3.2. Antibacterial Screening

The newly synthesized heterocyclic compounds were tested for their antimicrobial activity against Gram positive bacteria (*Staphylococcus aureus* and *Bacillus cereus*) and Gram negative bacteria (*Serratia marcesens* and *Proteus mirabilis*).

*Medium:* For all bacteria (Nutrient Medium), consisting of (g/L distilled water): peptone, 5 and meat extract, 3. pH was adjusted to 7.0. For solid media, 2% agar was added. All media were sterilized at 121 °C for 20 min.

#### 3.2.1. Agar Diffusion Method [24]

One mg of each of the newly synthesized compounds was dissolved in dimethyl sulphoxide (DMSO, 1 mL) then made up to 10 mL with sterile water to give a concentration of 100 μg/mL. A solution of the tested compounds was placed separately in the agar medium. The inhibition zones were measured after 24 h incubation.

#### 3.2.2. Filter Paper Disc-Diffusion Method [25]

Proper concentrations of microbial suspensions were prepared from one-day-old liquid stock cultures incubated on a rotary shaker (100 rpm). The mycelia were then subdivided by mechanical stirring at speed No. 1 for 30 min. Turbidity of bacteria was adjusted with a spectrophotometer at 350 nm to give an optical density of 1.0. Appropriate agar plates were aseptically surface inoculated uniformly by a standard volume (ca. 1 mL) of the microbial broth culture of the tested bacteria.Whatman No. 3 filter paper discs of 10 mm diameter were sterilized by autoclaving for 15 min at 121 °C. Test compounds were dissolved in 80% ethyl alcohol to give final concentration of 5 μg/mL. The sterile discs were impregnated with the test compounds (5 μg/disc). After the impregnated discs have been air dried, they were placed on the agar surface previously seeded with the organism to be tested. Discs were gently pressed with forceps to insure thorough contact with the media. Each test compound was conducted in triplicate. Plates were kept in the refrigerator at 5 °C for 1 h to permit good diffusion before transferring them to an incubator at 37 °C for 24 h.

## 4. Conclusion

Several new pyridines, pyrans, pyridazines, pyrazoles, isoxazoles and thiazines that contain a sulfonamido moiety were prepared using simple methods, their structures were proven by spectral methods and they were tested for their antibacterial activities. Most of these compounds showed promising activities against both Gram-positive and Gram-negative bacteria. These results are encouraging for synthesis of similar compounds in the near future.

## Figures and Tables

*Reaction conditions*: (**a**) acetylacetone/dioxane/Et_3_N/∆; (**b**) acetoacetanilde/dioxane/Et_3_N/∆; (**c**) ethyl acetoacetate, ethyl benzoylacetate, and/or ethyl caynoacetate/dioxane/Et_3_N/∆; (**d**) malononitrile/dioxane/Et_3_N/∆.

*Reaction conditions*: (**a**) hydrazine hydrate, Phenyl hydrazine, benzoyl hydrazine or thiosimicarbazide/dioxane/∆; (**b**) hydroxylamine hydrochloride/dioxane/AcONa/∆; (**c**) cyanoacetohydrazide/dioxane/Et_3_N/∆; (**d**) urea/EtOH/EtONa/∆; (**e**) thiourea or guanidine. HCl/EtOH/EtONa/∆.

**Table 1:** Antibacterial activity of the synthesized compounds: Agar diffusion method.

Compound No.	Gram positive		Gram negative	
	*Staphylococcus aureus*	*Bacillus cereus*	*Serratia marcesens*	*Proteus mirabilis*
**3**	++	++	++	++
**5**	-	-	+	+
**7**	++	++	+	+
**9a**	+	+	+	+
**9b**	+	-	+	+
**9c**	+	+	-	-
**11**	+	+	+	+
**12a**	+++	+++	+++	+++
**12b**	++	+++	++	++
**12c**	++	++	++	++
**12d**	+++	++	+	++
**13**	+	+	+	+
**16**	++	++	+++	+++
**17**	++	+++	+++	++
**18a**	+	+	+++	+
**18b**	++	++	+++	+++

The width of the zone of inhibition indicates the potency of antibacterial activity; (-) no antibacterial activity; (+) mild activity with the diameter of the zones equal to 0.5–0.8 cm, (++) moderate activity with the diameter of the zones equal to 1.1–1.2 cm; (+++) marked high activity with the diameter of the zones equal to 1.8–2.0 cm.

**Table 2:** Antibacterial activity of the synthesized compounds.

Comp. No.	Inhibition zone (mm)			
	Gram-positive		Gram-negative	
	*Staphylococcus aureus*	*Bacillus cereus*	*Serratia marcesens*	*Proteus mirabilis*
**3**	12	10	11	8
**5**	0	0	5	7
**7**	13	11	6	6
**9a**	3	5	3	4
**9b**	6	7	2	6
**9c**	6	6	8	5
**11**	5	7	7	8
**12a**	17	16	16	18
**12b**	15	12	13	11
**12c**	12	11	10	10
**12d**	16	12	7	11
**13**	7	7	5	8
**16**	13	11	16	17
**17**	17	12	18	14
**18a**	9	8	15	7
**18b**	12	13	17	15
**Chloram-phenicol®**	18	19	22	21
**Ampicilin® **	19	22	24	20

The sensitivity of microorganisms to the tested compounds is identified in the following manner: Highly sensitive = Inhibition zone 15–20 mm; Moderately sensitive = Inhibition zone: 10–15 mm; Slightly sensitive = Inhibition zone: 5–10 mm; Not sensitive = Inhibition zone: 0–5 mm; Each result represents the average of triplicate readings.
